# Supplementary material for: Evaluating a Single Domain Antibody Targeting Human PD-L1 as a Nuclear Imaging and Therapeutic Agent
Source: Cancers (Basel). 2019 Jun 21;11(6):872. doi: 10.3390/cancers11060872 (PMC6628009; doi:10.3390/cancers11060872)
Supplement: Supplementary file 1 [file cancers-11-00872-s001.pdf]

Supplementary Materials

Evaluating a Single Domain Antibody Targeting Human PD-L1 as a Nuclear Imaging and Therapeutic Agent

Katrijn Broos, Quentin Lecocq, Catarina Xavier, Jessica Bridoux, Tham T. Nguyen, Jurgen Corthals, Steve Schoonooghe, Eva Lion, Geert Raes, Marleen Keyaerts, Nick Devoogdt and Karine Breckpot

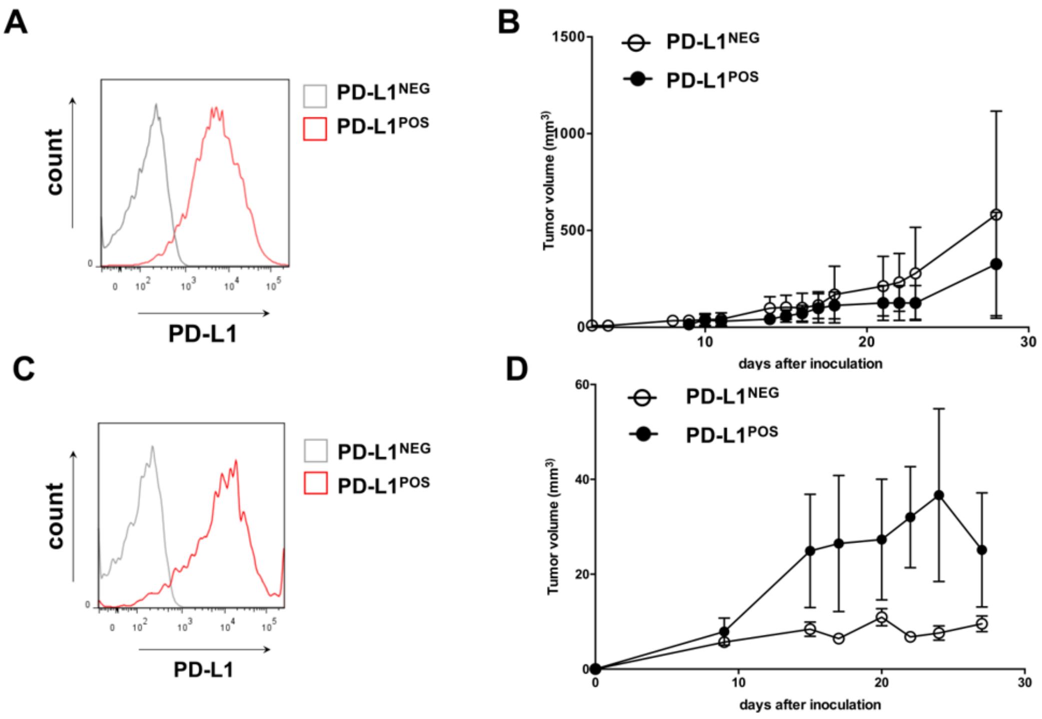

Figure S1. Characteristics of PD-L1^{POS} and PD-L1^{NEG} 624-MEL and MCF7 tumors. (**A**,**C**) Representative flow cytometry histograms showing PD-L1^{NEG} (grey) or engineered, PD-L1^{POS} (red) 624-MEL (**A**) or MCF7 cells (**C**), stained with an anti-PD-L1 mAb (n = 6). (**B**,**D**) Tumor growth of PD-L1^{POS} (black circle) or PD-L1^{NEG} (open circle) 624-MEL (**B**) or MCF7 cells (**D**) after subcutaneous (s.c.) implantation in athymic nude mice (n = 12).

© 2019 by the authors. Licensee MDPI, Basel, Switzerland. This article is an open access article distributed under the terms and conditions of the Creative Commons Attribution (CC BY) license (http://creativecommons.org/licenses/by/4.0/).
